# Supplementary material for: Intentional and unintentional non-adherence to social distancing measures during COVID-19: A mixed-methods analysis
Source: PLoS One. 2021 Aug 19;16(8):e0256495. doi: 10.1371/journal.pone.0256495 (PMC8376044; doi:10.1371/journal.pone.0256495)
Supplement: S1 Appendix. Survey questionnaire — (PDF) [file pone.0256495.s001.pdf]

COVID-19 Social Distancing

Information for Participants & Informed Consent

We would like to invite you to take part in a research study conducted by London Metropolitan University. This information sheet outlines the purpose of the study and provides a description of your involvement and rights as a participant. Please read the information and contact the researchers if you have any questions, the contact details are at the bottom of this form. You will then be able to decide if you are interested in taking part in the study.

1. What is the research about?

On the 23rd of March, the UK Government introduced lockdown mesures in response to the COVID-19 pandemic. This research study seeks to understand the relationships between demographics, values, beliefs, attitudes, and barriers experienced by the population in relation to social distancing and self-isolating during the pandemic.

2. Who can participate?

You are eligible to take part in this study if you are 18 years of age or older and a resident in the London boroughs of Islington, Haringey, Camden, Hackney, Barnet or Enfield.

3. Do I have to take part?

It is up to you to decide whether or not to take part. You do not have to take part if you do not want to. If you do decide to take part, continuing with this questionnaire will indicate your consent to participate. Everyone who completes the questionnaire and provides an email address will be entered into a prize draw with the chance of winning one of four £100 Aldi vouchers. If you do not wish to take part, please do not continue with this questionnaire.

4. What will my involvement be?

You are being asked to complete this questionnaire, which should take you between 5 and 10 minutes. In addition, we will ask if you would like to be contacted later to participate in a telephone interview about your experiences with social distancing and self-isolating. Again, participation in the telephone interview is completelty voluntary and does not affect your completion of the questionnaire and standing in the prize draw. If you are willing to participate in an interview you will be compensated for your time with a £20 Aldi voucher.

5. What will my information be used for?

Your information will exclusively be used for the research study described above. Your information and responses will not be used for any other purpose and will not be shared with anyone else. Only London Metropolitan University researchers will have access to your information and responses.

The results of this study may be published in scientific journals and at health and social care academic conferences. You will not be identified in any report or publication.

6. Will my taking part and my data be kept confidential? Will it be anonymised?

The records from this research study will be kept confidential. Only the researchers will have access to the data resulting from your responses. Your responses are anonymous – your name or any personal identifiers are not being requested. We are requesting your email address, so to notify you if you are a winner in the prize draw of four £100 Aldi vouchers. Your response will be given a code and stored separately and securely from a file containing your email address. Any hard copies of research information will be kept in locked files at all times.

7. What if I have a question or complaint?

If you have any questions regarding this study please contact the researchers, Dr. Stephen Hills (s.hills@londonmet.ac.uk) and Dr. Yolanda Eraso (y.eraso@londonmet.ac.uk).

If you have any concerns or complaints regarding the conduct of this research, please contact the Guildhall School of Business and Law's Research Ethics Review Panel (RERP) chair Eyob Mulat-Weldemeskel: e.mulat-weldemeskel@londonmet.ac.uk

I confirm that I have read and understood the information for the above study. I have had the opportunity to consider the information, ask questions and have had these answered satisfactorily ✱ *Required*

- Yes
- No

I understand that my participation is voluntary and that I am free to withdraw at any time without giving any reason ✱ *Required*

- Yes

- No

I understand that my personal involvement and my particular data from this study will remain strictly confidential. Only researchers involved in the study will have access ✱ *Required*

- Yes
- No

I understand that data resulting from my participation may be used for publications and academic conferences and that no personal information will be use in such cases ✱ *Required*

- Yes
- No

I confirm I am over 18 years old ✱ *Required*

- Yes
- No

I hereby fully and freely consent to participate in the study which has been fully explained to me ✱ *Required*

- Yes
- No

About You and Your Situation

What is your gender? ✱ *Required*

- ○ Male
- ○ Female
- ○ Other

What is your age in years? ✱ *Required*

Please enter a whole number (integer).

What is your ethnic group? ✱ *Required*

- ○ White
- ○ Mixed
- ○ Asian/Asian British
- ○ Black/Black British
- ○ Other

Is English your first language? ✱ *Required*

- ○ Yes
- ○ No

What is your religion? ✱ *Required*

- Christian
- Buddhist
- Hindu
- Jewish
- Muslim
- Sikh
- Other
- No religion

What is your highest level of education? ✱ *Required*

- No qualifications
- GCSEs or equivalent
- A Levels or equivalent
- Vocational/work-related qualification
- Bachelors degree
- Professional qualification
- Masters degree
- Doctoral degree

What is your postcode? ✱ *Required*

In the most recent general election, who did you vote for? ✱ *Required*

- Conservatives
- Labour

- Lib Dem
- Other
- Did not vote

Today, what is your employment status? ✱ *Required*

- Working as an employee in my normal place of work (not home)
- Working as an employee from home
- Self-employed or freelance in my normal place of work (not home)
- Self-employed or freelance from home
- A furloughed employee
- Unemployed
- Retired
- A student
- Looking after home or family
- Long-term sick or disabled
- Other

As defined by the UK Government, are you a key or essential worker? ✱ *Required*

- Yes
- No

As defined by the UK Government, do you have a medical condition that makes you more vulnerable to coronavirus (COVID-19) or are you pregnant? ✱ *Required*

- Yes
- No

What is your living situation? ✱ *Required*

- I live in a home I own
- I live in a home I rent
- I live in a rented room in a house of multiple occupancy

How many people live at your home? ✱ *Required*

Please enter a whole number (integer).

Do you live with someone who is considered to be more vulnerable to coronavirus (COVID-19), such as someone over 70 years old, someone who has a health condition which makes them more vulnerable, or someone who is pregnant? ✱ *Required*

- Yes
- No

Your Knowledge of Coronavirus (COVID-19)

Coronavirus (COVID-19) can only be caught from a person who has symptoms ✱ *Required*

- True
- False
- Don't Know

Coronavirus (COVID-19) can be spread when an infected person speaks ✱ *Required*

- True
- False
- Don't Know

The most common symptoms of coronavirus (COVID-19) are a dry cough, fever and loss of taste ✱ *Required*

- True
- False
- Don't Know

Coronavirus (COVID-19) can be caught by touching a contaminated surface and then your face ✱ *Required*

- True
- False
- Don't Know

Wearing latex gloves is more effective than hand washing at protecting against coronavirus (COVID-19) ✱ *Required*

- True
- False
- Don't Know

Social distancing restrictions permit me to go outside to walk my dog ✱ *Required*

- True
- False
- Don't Know

If I develop symptoms of coronavirus (COVID-19) I am still permitted to leave my house to collect a medical prescription ✱ *Required*

- True
- False
- Don't Know

If I develop symptoms of coronavirus (COVID-19), I should self-isolate for at least seven days ✱ *Required*

- True
- False
- Don't Know

If someone I live with develops symptoms of coronavirus (COVID-19), but I do not, I should

self-isolate for at least seven days ✱ *Required*

- True
- False
- Don't Know

If I was infected and there was no social distancing, it is estimated that a month later I would have infected over 400 people ✱ *Required*

- True
- False
- Don't Know

Your Social Distancing

In the past two weeks, how many times have you gone out for medication and come within two metres (approx. 3 steps) of someone (e.g., pharmacist, other customers) you don't live with? ✱ *Required*

Please enter a whole number (integer).

In the past two weeks, how many times have you gone out for groceries and come within two metres (approx. 3 steps) of someone (e.g. cashier, other shoppers) you don't live with? ✱ *Required*

Please enter a whole number (integer).

In the past two weeks, how many times have you gone out to enjoy parks or public spaces or for exercise and come within two metres (approx. 3 steps) of someone you don't live with? ✱ *Required*

Please enter a whole number (integer).

In the past two weeks, how many times have you broken social distancing rules to meet with extended family members that don't live with you? ✱ *Required*

Please enter a whole number (integer).

In the past two weeks, how many times have you broken social distancing rules to meet friends that don’t live with you? ✱ *Required*

Please enter a whole number (integer).

In the past two weeks, how many times have you gone out for reasons other than to work, to buy groceries, for medical reasons (e.g. to collect a prescription) to enjoy parks or public spaces or to exercise? ✱ *Required*

Please enter a whole number (integer).

Your Self-Isolating

Have you developed symptoms of coronavirus at any point (even if you believe that these symptoms were not in fact coronavirus)? ✱ *Required*

- Yes
- No

Your Self-Isolating

After developing symptoms of coronavirus, how many times did you leave your house (going to your garden does not count as leaving your house) for any reason within 7 days of developing symptoms? ✱ *Required*

Please enter a whole number (integer).

After developing symptoms of coronavirus, how many times did you have family or friends visit within 7 days of developing symptoms? ✱ *Required*

Please enter a whole number (integer).

Your Self-Isolating With Someone Vulnerable

Have you developed symptoms of coronavirus at any point (even if you believe that these symptoms were not in fact coronavirus) whilst living with someone considered more vulnerable to coronavirus (e.g. over 70, underlying health conditions, pregnant)? ✱ *Required*

- ○ Yes
- ○ No

Your Self-Isolating With Someone Vulnerable

Did you arrange for them to stay with friends or family for 14 days? ✱ *Required*

- Yes
- No

Were you able to maintain a distance of two metres (approx. 3 steps) from them for 7 days? ✱ *Required*

- Yes
- No
- Not relevant; we were able to arrange for them to stay with friends or family

Your Self-Isolating When Someone You Live With Has Symptoms

Has someone you live with developed symptoms of coronavirus at any point (even if they believe that these symptoms were not in fact coronavirus)? ✱ *Required*

- ○ Yes
- ○ No

Your Self-Isolating When Someone You Live With Has Symptoms

After someone you live with developed symptoms of coronavirus, how many times did you leave your house (going to your garden does not count as leaving your house) for any reason within 14 days of developing symptoms? ✱ *Required*

Please enter a whole number (integer).

After someone you live with developed symptoms of coronavirus, how many times did you have family or friends visit within 14 days of developing symptoms? ✱ *Required*

Please enter a whole number (integer).

Your Views

To what extent do you agree with the following statements? ✱ *Required*

	Entirely Disagree	Mostly Disagree	Somewhat Disagree	Neither Agree nor Disagree	Somewhat Agree	Mostly Agree	Entirely Agree
I take care of my own and my family's needs before I worry about the needs of others	☐	☐	☐	☐	☐	☐	☐
Before I act, I think about how my actions might have a negative effect on others	☐	☐	☐	☐	☐	☐	☐
I do what I want, regardless of what others want me to do	☐	☐	☐	☐	☐	☐	☐

Your Beliefs About Coronavirus (COVID-19)

To what extent do you agree with the following statements? ✱ *Required*

	Entirely Disagree	Mostly Disagree	Somewhat Disagree	Neither Agree not Disagree	Somewhat Agree	Mostly Agree	Entirely Agree
There is a good chance that I will get coronavirus (COVID-19)	☐	☐	☐	☐	☐	☐	☐
I do not worry a lot about getting coronavirus (COVID-19)	☐	☐	☐	☐	☐	☐	☐
I'm worried that if I caught coronavirus (COVID-19) my symptoms would be severe	☐	☐	☐	☐	☐	☐	☐

To what extent do you agree with the following statements? ✱ *Required*

	Entirely Disagree	Mostly Disagree	Somewhat Disagree	Neither Agree not Disagree	Somewhat Agree	Mostly Agree	Entirely Agree

I trust the UK Government in their response to COVID-19	☐	☐	☐	☐	☐	☐	☐
I trust that the Government is following the best scientific advice	☐	☐	☐	☐	☐	☐	☐
I trust the scientific advice that the Government has taken	☐	☐	☐	☐	☐	☐	☐

Your Beliefs About Social Distancing

To what extent do you agree with the following statements? ✱ *Required*

	Entirely Disagree	Mostly Disagree	Somewhat Disagree	Neither Agree not Disagree	Somewhat Agree	Mostly Agree	Entirely Agree
I will only leave my home for permitted reasons for as long as the lockdown measures are in place	☐	☐	☐	☐	☐	☐	☐
I will avoid contact with people I do not live with for as long as social distancing restrictions are in place	☐	☐	☐	☐	☐	☐	☐
I will not see friends or extended family in person for as long as the lockdown measures are in place	☐	☐	☐	☐	☐	☐	☐

To what extent do you agree with the following statements? ✱ *Required*

	Entirely Disagree	Mostly Disagree	Somewhat Disagree	Neither Agree not Disagree	Somewhat Agree	Mostly Agree	Entirely Agree
During lockdown, I do not need to leave my home if I don't want to	☐	☐	☐	☐	☐	☐	☐
When I go out for permitted reasons, I cannot stop others from coming within two metres of me	☐	☐	☐	☐	☐	☐	☐
I have responsibilities (e.g., work, childcare) for which I cannot avoid coming into contact with others that I do not live with	☐	☐	☐	☐	☐	☐	☐

To what extent do you agree with the following statements? ✱ *Required*

	Entirely Disagree	Mostly Disagree	Somewhat Disagree	Neither Agree not Disagree	Somewhat Agree	Mostly Agree	Entirely Agree

My family support staying at home and social distancing	☐	☐	☐	☐	☐	☐	☐
My friends are keen to meet up in person, despite the lockdown	☐	☐	☐	☐	☐	☐	☐
I see my neighbours keeping social distancing rules when they are out in my street	☐	☐	☐	☐	☐	☐	☐

Your Beliefs About Self-Isolating

To what extent do you agree with the following statements? ✱ *Required*

	Entirely Disagree	Mostly Disagree	Somewhat Disagree	Neither Agree not Disagree	Somewhat Agree	Mostly Agree	Entirely Agree
If I had symptoms, I would not need to leave my home for any reason	☐	☐	☐	☐	☐	☐	☐
If someone I live with had symptoms, I would not need to leave my home for any reason	☐	☐	☐	☐	☐	☐	☐
I have responsibilities (e.g., work, childcare) for which I would need to leave my home even if I had symptoms	☐	☐	☐	☐	☐	☐	☐

To what extent do you agree with the following statements? ✱ *Required*

	Entirely Disagree	Mostly Disagree	Somewhat Disagree	Neither Agree not Disagree	Somewhat Agree	Mostly Agree	Entirely Agree

My immediate family would not go out for any reason if they had coronavirus symptoms	☐	☐	☐	☐	☐	☐	☐
My friends would not go out for any reason if they had coronavirus symptoms	☐	☐	☐	☐	☐	☐	☐
My extended family would not go out for any reason if they had coronavirus symptoms	☐	☐	☐	☐	☐	☐	☐

Your Support

To what extent do you agree with the following statements? ✱ *Required*

	Entirely Disagree	Mostly Disagree	Somewhat Disagree	Neither Agree not Disagree	Somewhat Agree	Mostly Agree	Entirely Agree
During lockdown, there is a special person who is around when I am in need	☐	☐	☐	☐	☐	☐	☐
During lockdown, there is a special person with whom I can share joys and sorrows	☐	☐	☐	☐	☐	☐	☐
During lockdown, my family really tries to help me	☐	☐	☐	☐	☐	☐	☐
During lockdown, I get the emotional help & support I need from my family.	☐	☐	☐	☐	☐	☐	☐

During lockdown, I have a special person who is a real source of comfort to me	☐	☐	☐	☐	☐	☐	☐
During lockdown, my friends really try to help me	☐	☐	☐	☐	☐	☐	☐
During lockdown, I can count on my friends when things go wrong	☐	☐	☐	☐	☐	☐	☐
During lockdown, I can talk about my problems with my family	☐	☐	☐	☐	☐	☐	☐
During lockdown, I have friends with whom I can share my joys and sorrows	☐	☐	☐	☐	☐	☐	☐

During lockdown, there is a special person in my life who cares about my feelings	☐	☐	☐	☐	☐	☐	☐
During lockdown, my family is willing to help me make decisions	☐	☐	☐	☐	☐	☐	☐
During lockdown, I can talk about my problems with my friends	☐	☐	☐	☐	☐	☐	☐

During lockdown, are you getting the help you need with your financial situation from UK Government schemes? ✱ *Required*

- ○ Yes
- ○ No
- ○ Help Not Needed

During lockdown, are you getting the help you need with your housing situation from UK Government schemes? ✱ *Required*

- ○ Yes
- ○ No
- ○ Help Not Needed

During lockdown, are you getting the help you need from local community services (e.g., your council, voluntary or charity organisations)? ✱ *Required*

- ○ Yes
- ○ No
- ○ Help Not Needed

# Email Address & Future Involvement

If you are randonly selected to win a £100 Aldi voucher, we need your email address to notify you. Providing your email address is optional.

What is your email address? *Optional*

More info

Please enter a valid email address.

We would like to conduct follow-up telephone interviews with certain individuals. This involvement is optional and does not impact your standing in the proze draw for the £100 Aldi vouchers.

I am willing to be contacted by email to schedule a follow up telephone interview for which I will be compensated with a £20 Aldi voucher ✱ *Required*

- Yes
- No

Thank You

This is the end of the questionnaire.

Thank you for completing it and for participating in this research project.

If you are randomly selected to win a £100 Aldi voucher we will notify you via email.
