# Supplementary material for: Intentional and unintentional non-adherence to social distancing measures during COVID-19: A mixed-methods analysis
Source: PLoS One. 2021 Aug 19;16(8):e0256495. doi: 10.1371/journal.pone.0256495 (PMC8376044; doi:10.1371/journal.pone.0256495)
Supplement: S2 Appendix — (PDF) [file pone.0256495.s002.pdf]

INTERVIEW GUIDE

1. How would you describe your experience during the ‘lockdown’?

2. What measures were more difficult for you to comply with: Staying at home or keeping 2 mts. distancing when going out? [expand if necessary]

3. Overall, how would you consider the level of your compliance to lockdown measures?

Theory of Planned Behaviour (TPB): Perceived behavioural control

4. How easy or difficult was for you to keep 2mts. distance from people? [probe - what were the main difficulties?]
5. During the lockdown, did you feel you could stay at home for as long as you wanted, or you felt you didn’t have a choice and had to leave your home for some reasons? [probe -Can you specify the reasons?]
6. What do you think would have make it easy or easier for you to stay at home during the lockdown? Can you think of anything in particular?

TPB Social norms:

7. Were there particular individuals or groups of people that has supported or approved you to stay at home and only going out for permitted reasons? Did anyone disapprove?

Health Belief Model: Perceived susceptibility/anticipated severity

8. Do you feel you are likely to catch Covid-19? If so, for which reasons?
9. If you were to catch Covid-19, what health consequences do you feel the disease could have for you? [probes: physical, mental health, work, finance, impact on the family/ household, access to food/ medications].

Socio Ecological Model (SEM): Housing

11 Intro- tell me about your housing arrangements: How many people live with you? [number] Is anybody considered vulnerable or at higher risk for Covid-19?

12 Given your housing arrangements/and rooms available: Did you have any concerns about you, a family member living with you, or a housemate becoming infected with Covid-19? [prob- expand based on the answer, Key worker, shared accommodation?]

13 Did someone living with you have to self-isolate because of symptoms? – If answer yes: What can you tell me about the self-isolating experience at your home? [probes: Could you keep distance, any concerns?]

SEM: Support

14 During lockdown, what was the main source of emotional support that helped you in staying at home? [probes: friends, family, partner, community groups, faith groups].

15 Did you need and material support to help you stay at home? From whom? [expand based on groups above].

SEM: Finances [related to employment]

16 Did the lockdown affect you financially? In which ways?
17 Did a change in your financial circumstances have an impact in your ability to stay at home and social distancing [keep 2mts]?

TPB: Intentions

18 How likely are you to comply with staying at home /keeping social distancing measures if reinstated in the future?

Trust in Gov:

19 Have your intentions to comply with the Government 'lockdown' measures changed since the pandemic started? [probes: timing - during full & easy of lockdown, trust in Government, meaning of 'change']
20 Did your attitude to follow social distancing rules change after any specific political event? If so, in which ways your attitude changed? [probes: Cummings scandal; presentation of data; timing for easing the lookdown measures]

Final questions

21 In your view, is there anything that could have been done better to help you comply with the lockdown measures? [probes: identify if by Government, by council, or other stakeholders]
22 Do you have anything else you would like to add?
