# Supplementary material for: Intentional and unintentional non-adherence to social distancing measures during COVID-19: A mixed-methods analysis
Source: PLoS One. 2021 Aug 19;16(8):e0256495. doi: 10.1371/journal.pone.0256495 (PMC8376044; doi:10.1371/journal.pone.0256495)
Supplement: S3 Appendix — (DOCX) [file pone.0256495.s003.docx]

**S1 Table. Average infringements by category of categorical explanatory variable**

| **Explanatory variables** | **All infringements** | | **Intentional infringements** | |
| --- | --- | --- | --- | --- |
|  | **Mean** | **S.D.** | **Mean** | **S.D.** |
| Sample | 10.77 | 9.36 | 2.59 | 4.62 |
| **Demographic factors** |  |  |  |  |
| Gender |  |  |  |  |
| Female | 10.73 | 9.01 | 2.76 | 5 |
| Male | 11.27 | 9.45 | 2.57 | 4.56 |
| Other | 5 | 5.33 | 1.5 | 2.74 |
| Ethnicity |  |  |  |  |
| White | 10.9 | 9.4 | 2.64 | 4.63 |
| BAME | 10.01 | 9.16 | 2.33 | 4.59 |
| Language |  |  |  |  |
| English as first language | 10.78 | 9.56 | 2.6 | 4.75 |
| English not as first language | 10.73 | 8.17 | 2.55 | 3.83 |
| Religion |  |  |  |  |
| No religion | 10.65 | 9.02 | 2.52 | 4.38 |
| Christian | 11.34 | 10.06 | 2.94 | 5.17 |
| Buddhist | 10 | 8.09 | 3.67 | 5.36 |
| Hindu | 4.33 | 3.79 | .67 | 1.16 |
| Jewish | 11.38 | 10.63 | 3.23 | 5.8 |
| Muslim | 8.57 | 7.92 | 2.07 | 3.63 |
| Sikh | 9.5 | 9.19 | 0 | .00 |
| Other | 10.38 | 9.78 | .85 | 1.74 |
| Highest qualification obtained |  |  |  |  |
| No qualifications | 16.62 | 14.33 | 3.92 | 5.38 |
| GCSEs or equivalent | 10.9 | 7.88 | 3.73 | 5.17 |
| A Levels or equivalent | 8.8 | 7.25 | 2.27 | 3.99 |
| Vocational / work-related qualification | 9.05 | 9.17 | 1.55 | 2.86 |
| Bachelor’s degree | 11.16 | 10.17 | 2.69 | 4.66 |
| Professional qualification | 9.37 | 7.81 | 2 | 3.66 |
| Master’s degree | 10.84 | 8.88 | 2.68 | 5.17 |
| Doctoral degree | 12.95 | 10.21 | 2.87 | 4.6 |
| Employment Status |  |  |  |  |
| Long-term sick or disabled | 8.86 | 10.24 | 1.32 | 2.76 |
| Retired | 7.88 | 10.4 | 3.09 | 6.36 |
| Working as an employee from home | 10.85 | 9.64 | 2.31 | 4.58 |
| Self-employed or freelance from home | 10.39 | 8.04 | 2.03 | 4 |
| Looking after home or family | 9.59 | 6.76 | 2.97 | 4.15 |
| Unemployed | 10.17 | 8.29 | 2.11 | 3.76 |
| A furloughed employee | 12.97 | 10.19 | 3.69* | 5.07 |
| A student | 11.95 | 8.78 | 3.85 | 4.67 |
| Working as an employee in normal place of work (not home) | 12.03 | 8.5 | 2.37 | 4.07 |
| Self-employed or freelance in normal place of work (not home) | 15.06 | 9.26 | 5.81* | 5.28 |
| Other | 9.39 | 9.34 | 2.48 | 4.15 |
| Key Worker status |  |  |  |  |
| Not key worker | 10.57 | 9.31 | 2.54 | 4.55 |
| Key worker | 11.45 | 9.53 | 2.79 | 4.64 |
| **Housing factors** |  |  |  |  |
| Housing situation |  |  |  |  |
| Live in own home | 9.85 | 9.25 | 2.67 | 4.92 |
| Live in rented home | 11.64* | 9.56 | 2.47 | 4.39 |
| Live in rented room of multiple occupancy house | 12.06 | 8.94 | 2.66 | 3.99 |
| Living with a vulnerable person |  |  |  |  |
| Living with person of vulnerable health status | 9.36 | 9.1 | 2.25 | 4.64 |
| Not living with person of vulnerable health status | 11.03 | 9.39 | 2.66 | 4.62 |
| **Health factors** |  |  |  |  |
| Vulnerable health |  |  |  |  |
| Vulnerable | 7.29 | 8.84 | 1.75 | 4.16 |
| Not vulnerable | 11.38* | 9.32 | 2.74* | 4.68 |
| COVID-19 Symptoms |  |  |  |  |
| Not had | 10.6 | 9.53 | 2.52 | 4.73 |
| Had | 11.17 | 8.97 | 2.76 | 4.37 |
| **Political factors** |  |  |  |  |
| 2019 General election |  |  |  |  |
| Voted for Government | 11 | 10.65 | 3.7 | 5.83 |
| Did not vote for Government | 10.75 | 9.23 | 2.48 | 4.48 |
| Lockdown phase |  |  |  |  |
| Total lockdown | 10.47 | 9.21 | 2.08 | 4.12 |
| Overlap of total and first relaxation | 11.12 | 9.07 | 2.58 | 4.36 |
| First relaxation | 10.71 | 10.06 | 3.4* | 5.56 |
| **Social factors** |  |  |  |  |
| Financial support |  |  |  |  |
| Getting financial support if needed | 10.91 | 9.53 | 2.68 | 4.85 |
| Not getting financial support if needed | 10.21 | 8.68 | 2.26 | 3.56 |
| Community support |  |  |  |  |
| Getting community support if needed | 10.88 | 9.46 | 2.64 | 4.72 |
| Not getting community support if needed | 9.96 | 8.58 | 2.23 | 3.8 |

*Statistically significantly higher infringements

**S2 Table. Associations between numerical explanatory variables and infringements**

| **Explanatory variables** | **All infringements** | | **Intentional infringements** | |
| --- | --- | --- | --- | --- |
|  | **Correlation** | **Sig.** | **Correlation** | **Sig.** |
| **Demographic factors** |  |  |  |  |
| Age | -.104* | .006 | .053 | .169 |
| Deprivation | -.045 | .243 | .015 | .702 |
| **Housing factor** |  |  |  |  |
| Number of people living with | .047 | .216 | .044 | .257 |
| **Health factor** |  |  |  |  |
| Perceived susceptibility | .067 | .079 | .061 | .114 |
| **Political factor** |  |  |  |  |
| Trust in Government | -.034 | .377 | -.033 | .389 |
| **Psychological factors** |  |  |  |  |
| COVID-19 and social distancing knowledge | -.062 | .106 | -.08* | .036 |
| Social responsibility | -.097* | .012 | -.147* | .000 |
| Self-interest | .057 | .141 | .143* | .000 |
| Intention to socially distance | -.337* | .000 | -.409* | .000 |
| Control over leaving the house | -.208* | .000 | -.1* | .009 |
| Control over others’ distancing | -.217* | .000 | -.09* | .019 |
| Control over responsibilities | -.204* | .000 | -.13* | .001 |
| Normative pressure from family | -.117* | .002 | -.189* | .000 |
| Normative pressure from friends | -.222* | .000 | -.21* | .000 |
| Normative pressure from neighbours | -.011 | .768 | .044 | .247 |
| **Social Factors** |  |  |  |  |
| Support from a special person | .032 | .411 | .001 | .978 |
| Support from family | .023 | .554 | -.001 | .987 |
| Support from friends | .097* | .011 | .06 | .117 |

*Statistically significant simple association
